# Spinal Fractures during Touristic Motorboat Sea Cruises: An Underestimated and Avoidable Phenomenon

**DOI:** 10.3390/jcm12041426

**Published:** 2023-02-10

**Authors:** Adrien Thomas May, Nicolas Bailly, Aurore Sellier, Valentin Avinens, Maxime Huneidi, Mikael Meyer, Lucas Troude, Pierre-Hugues Roche, Henry Dufour, Arnaud Dagain, Pierre-Jean Arnoux, Kaissar Farah, Stéphane Fuentes

**Affiliations:** 1 Service de Neurochirurgie, Hôpital de la Timone, Marseille, Assistance Publique Hôpitaux de Marseille, 13005 Marseille, France; 2 Service de Neurochirurgie, Hôpital Nord, Marseille, Assistance Publique Hôpitaux de Marseille, 13005 Marseille, France; 3 Laboratoire de Biomécanique Appliquée, UMRT24 IFSTTAR—Université de la Méditerranée, 13005 Marseille, France; 4 Hôpital d’Instruction des Armées, 83000 Toulon, France

**Keywords:** sea, thoracolumbar, spinal, fractures, motorboat

## Abstract

Purpose: Each summer, many vacationers enjoy the Mediterranean Sea shores. Among the recreational nautical activities, motorboat cruise is a popular choice that leads to a significant number of thoracolumbar spine fractures at our clinic. This phenomenon seems to be underreported, and its injury mechanism remains unclear. Here, we aim to describe the fracture pattern and propose a possible mechanism of injury. Methods: We retrospectively reviewed the clinical, radiological, and contextual parameters of all motorboat-related spinal fracture cases during a 14-year period (2006–2020) in three French neurosurgical level I centers bordering the Mediterranean Sea. Fractures were classified according to the AOSpine thoracolumbar classification system. Results: A total of 79 patients presented 90 fractures altogether. Women presented more commonly than men (61/18). Most of the lesions occurred at the thoracolumbar transition region between T10 and L2 (88.9% of the levels fractured). Compression A type fractures were seen in all cases (100%). Only one case of posterior spinal element injury was observed. The occurrence of neurological deficit was rare (7.6%). The most commonly encountered context was a patient sitting at the boat’s bow, without anticipating the trauma, when the ship’s bow suddenly elevated while crossing another wave, resulting in a “deck-slap” mechanism hitting and propelling the patient in the air. Conclusions: Thoracolumbar compression fractures are a frequent finding in nautical tourism. Passengers seated at the boat’s bow are the typical victims. Some specific biomechanical patterns are involved with the boat’s deck suddenly elevating across the waves. More data with biomechanical studies are necessary to understand the phenomenon. Prevention and safety recommendations should be given before motorboat use to fight against these avoidable fractures.

## 1. Introduction

Different kinds of spine fractures related to sea and lake activities have been reported [1,2,3,4,5,6,7]. In each situation, a specific injury mechanism describes a proper pattern of the lesion. For instance, cervical fractures resulting from shallow-water headfirst dives lead to severe injuries with potential deleterious neurological outcomes [8]. Cliff jumpers generally present with thoracolumbar burst fractures from high compressive energy delivered to the calcaneum when entering the water [2]. In addition, motorized activities are also culprits of various fracture types, with the most commonly studied injury involving patients using personal watercrafts (jet skis) and presenting with polytraumatic lesions involving multiple bones [3,9,10]. Military ships or speedboat catastrophes also present high-energy traumatic mechanisms with various associated limb fractures [11].

The biomechanics of thoracolumbar fractures have been well-described. Different patterns of trauma have been analyzed resulting in different fracture types [12,13]. Typically, AOSpine A type fractures result from a combination of high compression forces associated with some degrees of anterior flexion. Few case reports and only one series have reported compression fractures originating from wavy boat rides [14,15,16,17,18]. These unexpected fractures occur in relatively calm conditions and are typically a surprise to boat occupants. This raises the suspicion that the biomechanics behind these boat fractures are different compared with traditional thoracolumbar fractures and suggests that their mechanism remains unclear, ultimately hindering their prevention.

The objective of this study is to report our observations in the management of these thoracolumbar fractures with emphasis on fracture types, context, and proposing a possible biomechanism responsible for this specific entity.

## 2. Methods

### 2.1. Study Design and Patients

This retrospective multicenter observational study was conducted between January 2014 and December 2020 at the “Assistance Publique des Hôpitaux de Marseille” comprising two neurosurgical centers (Hôpital Nord and Hôpital La Timone) and between January 2006 and December 2020 at the “Sainte-Anne Military Hospital”, Toulon.

All patients with a spinal thoracolumbar injury resulting from a touristic motorboat accident were included. We collected data on patient demographics (age, sex, body mass index (BMI)), comorbidities, neurological status, fracture level, fracture type according to the AOSpine Thoracolumbar Spine Injury Classification [19]), and other associated injuries. The exclusion criterion was trauma unrelated to touristic motorboat activities. Radiological evaluation included systematic preoperative examinations with spine CT scan and MRI. Fracture levels and types [19] were reported. Precise contextual information about the injury mechanism, type of boat, and sea conditions during the accident was obtained through medical records and by retrospectively calling the patients to the fracture management.

### 2.2. Statistical Evaluation

The correlation between parameters was searched using Pearson’s chi-square correlation test. A *p* value < 0.05 was considered statistically significant. Statistical analysis was obtained with GraphPad Prism (GraphPad Software, Inc., California, CA, USA).

## 3. Results

A total of 82 patients met the inclusion criteria. Three patients were excluded from the study because of a personal watercraft (jet ski) accident with a different injury mechanism (high-velocity polytrauma). A total of 79 patients were included in the final database comprising 90 thoracolumbar fractures. No associated cervical lesion was found.

### 3.1. Demographics (Table 1)

**Figure 1:**
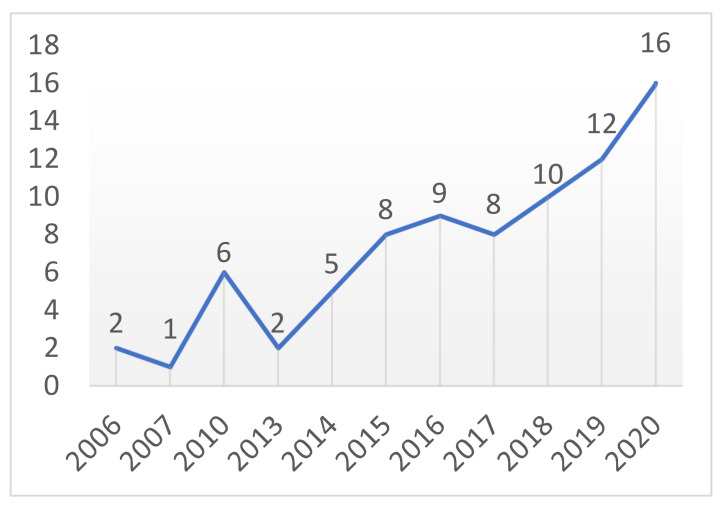
Evolution of retrieved patients per year.

The cohort included 61 women and 18 men. The mean age was 45 years (standard deviation (SD) 15), mean weight was 64.7 kg (SD 12.8), mean height was 167.9 cm (SD 9.1), and BMI was 22.8 kg/m^2^ (SD 3.4). Three patients had confirmed osteoporosis, and one had hypothyroidism. Two patients presented with associated upper-limb fracture. The incidence tended to increase over the years (Figure 1). Using the AOSpine classification, neurologic impairment was seen in 6 cases: 1 bladder incontinence (N3), 3 radiculopathy (N2), and 2 spinal cord compression (N3) [19]. Five neurological deficits were only temporary deficits, and one patient had long-term sequelae.

**Table 1:** Demographical data.

	Total
Patients	79
Women/Men	61/18
Age mean (SD)	45 year (15)
Weight mean (SD)	64.7 kgs (12.8)
Height mean (SD)	167.9 cm (9.1)
BMI mean (SD)	22.84 kg/m^2^ (3.4)
Neurologic impairment (%)	6 (7.6)
Fractures per patient (%)	
1 level	70 (88.6)
2 levels	7 (8.9)
3 levels	2 (2.5)
Total fractures (%)	90 (100)
T7	1 (1.1)
T8	0 (0)
T9	1 (1.1)
T10	2 (2.2)
T11	6 (6.7)
T12	21 (23.3)
L1	38 (42.2)
L2	13 (14.4)
L3	8 (8.9)

### 3.2. Fracture Characteristics

**Figure 2:**
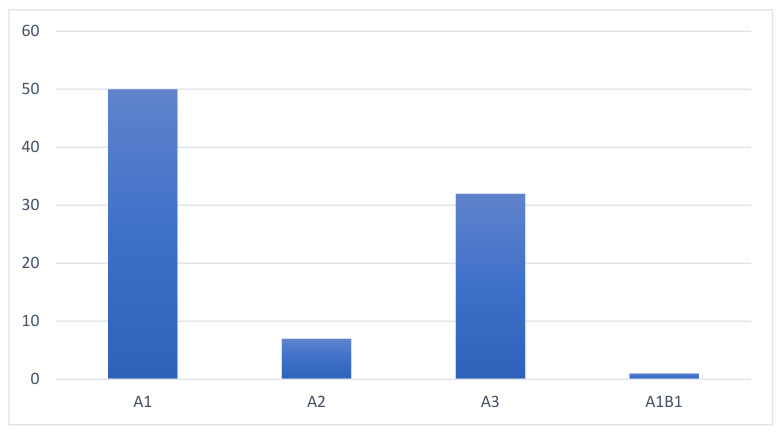
Fracture repartition according to the AOSpine classification system [19].

A type fractures according to the AO classification system occurred in all cases, with a single case being classified as B1 with A1 subtype. A total of 50 fractures were classified as type A1, 7 as type A2, and 32 as type A3 (Figure 2).

**Figure 3:**
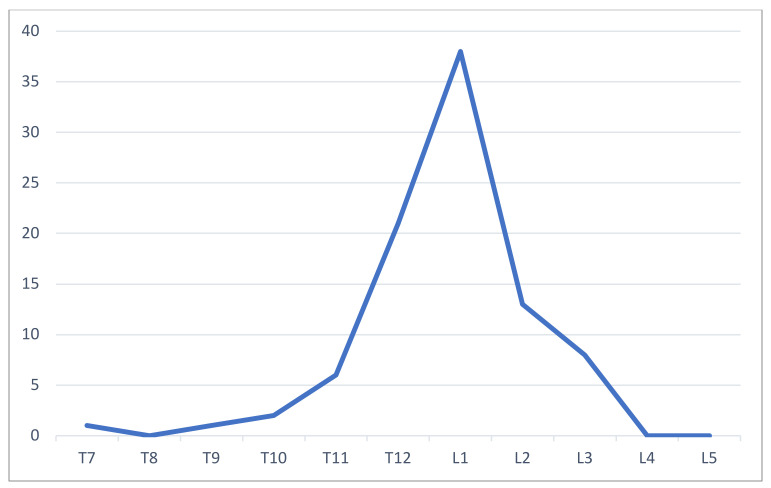
Fracture distribution along the spine levels.

A total of 70 patients presented a one-level fracture, 7 involved a two-level lesion, and 2 involved a three-level fracture. Patients with multiple levels involved did not show any specific comorbidity such as hypothyroidism or osteoporosis. The thoracolumbar junction (T12–L1) was the most affected site: T12 was injured in 21 cases (23.3%) and L1 in 38 cases (42.2%). The next most injured vertebra was L2 in 13 patients (Figure 3). Approximately 88.9% of the fractured levels were comprised between T10 and L2.

The differences between demographical factors (age and sex), the BMI, and the fracture type or location are presented in Table 2, Table 3 and Table 4. Table 2 shows that the majority of fractures occurred in the female population (77.8%); however, there were no differences among the type. There was no apparent statistical difference in their repartition between sexes (*p* = 0.072).

Eighty percent of injuries occurred between the ages of 21 and 60 years. A1 was the most common fracture type, followed by A3, in all categories. There was no statistical difference in fracture repartition between ages (*p* = 0.454) as seen in Table 3.

The fracture distribution according to the BMI was explored. More than half of the fractures were in patients with a normal BMI (51 fractures, 56.7%). The fracture distribution was similar, with A1 and A3 still being the most represented in the same proportions in each group. There was no statistical difference between the BMI groups (*p* = 0.59). A total of 14 patients had missing data (weight or height) for which no BMI could be calculated (Table 4).

### 3.3. Context of Injury

Of the 79 patients, 71 could be contacted by phone and were able to describe the accident’s circumstances. The most common situation when the injury occurred was on a semi-rigid ship (53.5%), with the patient sitting (97%) at the boat’s bow (85.9%). After crossing an unanticipated wave (73%) coming from another boat’s wake (77.5%), the patient was projected in the air by a “hit” from the boat’s deck. The patient landed on their bottom (88.7%). In the majority of the cases, the sea was calm or with small waves (68%). Our series did not have a typical boat speed (considered slow in 22.5%, moderate in 31%, and high in 32.4%). Almost all cruises were touristic (93%). More detailed information can be found in Table 5.

## 4. Discussion

Spinal fractures related to recreational boating seem common all along the Mediterranean seashores. This phenomenon appears, however, to be underreported with only a few articles available in the literature. Sellier reported the largest series of 26 vertebral compression fractures occurring in the thoracolumbar region in the French Riviera [18]. Maempel also reported a series of 21 patients showing similar patterns of thoracolumbar fracture in Malta [17]. Other reports have also described the phenomenon as isolated cases [15,16]. After pooling our data from three level I neurosurgical centers, we confirmed the pattern of the lesion and further analyzed the mechanisms. Some similar “bump” fractures have also been described in city bus rides [20].

### 4.1. Risk Factors

In our series, the sex ratio was clearly in favor of women. A spontaneous primary explanation would be the higher prevalence of osteoporosis in this population [21]. Nevertheless, the evaluation of comorbidities in this series found only 3 cases of confirmed densitometric osteoporotic patients. This entity is probably underestimated here; however, it does not appear as a clear factor of risk. An alternative theory could be the prevalence of male captains driving the boat, while their spouses/friends being at the boat’s bow, consequently carrying more significant risk.

The age of presentation (a mean of 45 years with an SD of 15 years) also speaks against the association between osteoporosis and fractures. Osteoporotic fractures are generally seen in older populations than in this series [21]. The height, weight, and BMI were within normal ranges, and no correlation with fracture occurrence or severity was found. This reinforces the impression that boat-related fractures concern the whole population, with no specific subpopulation being more at risk.

In our study, the incidence of boat-related fracture has risen from 2006 to 2020 (Figure 1). The data between 2006 and 2013 were given from only one neurosurgical center (St Anne). The patient data from the two others centers (La Timone and Hôpital Nord) were obtained since 2014 only. Before this date, patient records were non-informatized and non-available. Furthermore, this progressing incidence is probably also explained by the increased awareness of this clinical entity during the time. Supporting data regarding the rising number of motorboat accidents leading to more fractures are insufficient.

### 4.2. Repartition and Fracture Type

A1 and A3 fractures (single endplate fracture with or without posterior wall involvement) represented 82 patients (91%). Most of the fractured levels were encountered at the thoracolumbar transition zone. T12 and L1 were the most attained levels and represented 21 and 38, respectively, out of the total 90 fractures. No cervical fractures were found, and multiple levels were rarely involved. When multiple levels were fractured, the results of our analysis did not show any specific risk based on the data that were collected. We were also not able to relate fracture patterns with the context of the traumatic event. As is commonly seen in practice and widely reported, the thoracolumbar junction was most commonly involved. The reason for this is likely related to the fact that the thoracic spine has a limited range of motion because the ribs function as a natural cast and the coronally oriented facets limit its movement. Schematically, it behaves as a rigid stick. In contrast, the lumbar spine maintains higher motion amplitudes. The upper part of the lumbar region is less resistant to compressive forces than the lower levels [22]. When compression is applied, the energy delivered is more prone to fracture from T10 to L2. This mechanism is supported as AOSpine type A fractures result from compression or flexion/compression mechanism [12]. In a finite-element model, Li and Guo showed that flexion added to the compressive force of traumatism was likely to generate a superior endplate fracture [23]. These elements argue for a flexed position during the accident, with the patient likely sitting on the boat leaning forward.

### 4.3. Suspected Mechanisms

Considering the contextual data, most injured patients were localized at the boat’s bow in a sitting position. The ship crossed a sudden, unexpected wave, resulting in a shock and the concomitant spinal trauma. This provides elements to evaluate the effect of muscle relaxation in the context of a sudden and unexpected trauma.

Two biomechanical theories can be considered from the described situation. The first suggests that the fracture occurred by the boat’s deck suddenly hitting the patient. This phenomenon is known as the “deck-slap” injury model proposed by Sellier. It shares similar mechanisms with a blast under a military vehicle, pushing its deck against the patient [24]. However, in our series, no patient had the typical lower extremity fractures encountered during military vehicle explosions [25]. This is probably partially explained by the different positions of a patient in a car and on a boat. In boat-related fractures, the patient is generally sitting with an anterior flexion, which is less likely to be seen with vehicle occupants maintained by the seatbelt. This difference is important as flexion added to compression results in higher compressive loads in the vertebras [22]. The trauma mechanism seems, however, distinctive in boat fractures as the injury energy appears to be significantly lower on the water, without any blast deflagration.

A second theory hypothesizes that the patient is propelled in the air by the bow movement due to the crossing wave. Following this model, the fracture occurs when the patient drops on the boat’s deck. In this series, 89% of the victims landed on their bottom. However, this model is less convincing as young and healthy patients do not commonly present a fracture after a fall from their height. In this study, the boat’s behavior and the effect of the wave type have not been properly modeled. The boat’s bow remains a particular location where patients were injured, suggesting a specific acceleration/deceleration pattern. In 1983, Wiker and Miller studied the accelerations resulting from different boats crossing two types of wakes [26]. They compared mono- and tri-hull boat designs without evidencing differences in terms of acceleration forces. Our series also did not indicate a particular boat being more at risk between rigid and semi-rigid hull. In contrast, the higher the wave and the faster the boat’s speed in Wiker and Miller’s model, the higher the acceleration. A correlation with spinal fracture occurrence was not evident as their experiment showed results below the thresholds in terms of acceleration and duration accepted to generate a spinal lesion. However, it is essential to note that a simple anthropomorphic model was used without considering spinal flexion during trauma (which leads to more compressive forces) and a possible relation with muscle relaxation. The boater is surprised because the accident occurs during calm cruises (73% of the patients in this series were surprised vs. 21% of them anticipated the wave). We can argue that they were seated relaxed on the boat. Potential anticipation could have led to contraction of the paraspinal musculature and, thus, spinal protection. Its effect should, however, be studied in more detail using larger studies.

Practically, given the data we obtained on the context of these injuries, we asked ourselves how prevention and limitation of their occurrence could be improved. We recognized three main directions to work with: (1) Raise awareness in the general population about the unexpected dangers of touristic motorboating. The classic context leading to the fracture needs to be known by the boaters (bow-sitting in an anterior flexed position, non-anticipation of a crossing wave, etc.). We consequently contacted our regional health agencies to discuss prevention measures. (2) Awareness of this phenomenon should be communicated to the boat’s manufacturers. After discussing with boat rental companies, it is clear that this vertebral fracture pattern has been encountered for a long time. If more information regarding this phenomenon was brought to the boat’s manufacturers, safety prevention could be incorporated in the boat’s design (e.g., development of shock-absorbing cushions, technical modification of the boat’s bow to reduce acceleration/deceleration forces, etc.). (3) Finally, we think that this entity should be discussed and learned during boating license training and examination. This would be a very effective way of limiting these fractures by teaching the captains how to diminish the risks through recognizing the classical injury context and adopting some adjusted driving skills.

#### Limitations

There are some limitations to this study. First of all, its retrospective nature potentially adds to the lack of precise data. A selection bias may also exist with patients with the more severe and urgent fractures being admitted to the hospitals. Simple bone bruises or light pain fractures could have been omitted leading to an overrepresentation of severe fractures. Likewise, this may underestimate the incidence and prevalence of this injury.

Another limitation is the duration of the study, with some patients called back almost 15 years after the trauma. Specifically, the contextual data could suffer some imprecisions from that aspect resulting in potential recall bias.

Treatment options have not been described in this paper as a matter of simplification and because of the different strategies in each center; however, there is no indication that treatment for these fractures should be any different than the standard of care that is provided for other compression fractures. Having said this, prevention remains the cornerstone of these spinal injuries, and, therefore, regional health agencies have been contacted and notified of our results.

## 5. Conclusions

This series underlines from several angles the importance of spinal compression fractures related to tourism boat excursions. Their occurrence seems to be underestimated, and prevention can be easily made by public health officers and boating rental companies. The risk of vertebral fracture is probably poorly known by the boaters and their captains. Its biomechanism is not well-understood yet by clinicians, but its further study and comprehension can improve the overall safety of individuals undertaking boating activities. This study highlights the fact that this classical traumatic condition involves calm sea, touristic nautical trips, and patients seated at the boat’s bow. Overrepresentation of women may be a selection bias, and both sexes appear to be at risk. No specific comorbidities can be identified as a risk factor for influencing the occurrence of fractures, and their occurrence is probably based more on the specific biomechanism involving either a sudden elevation of the boat’s deck with a resulting “deck-slap” compression occurring from below, or sufficient propulsion of the patient in the air that drops back on the boat’s deck.

## Figures and Tables

**Table 2:** Fracture distribution according to sex.

Sex	Men	Women
Total fractures (%)	20 (22.2)	70 (77.8)
A1	10	40
A2	4	3
A3	6	26
B1A1	0	1

**Table 3:** Fracture distribution in relation to age.

Age (Years)	0–20	21–40	41–60	>60
Total fractures (%)	6 (6.7)	33 (36.7)	39 (43.3)	12 (13.3)
A1	5	15	24	6
A2	0	3	4	0
A3	1	15	11	5
B1A1	0	0	0	1

**Table 4:** Fracture distribution related to BMI.

BMI (kg/m^2^)	<18	18–25	>25	Unknown BMI
Total fractures (%)	8 (8.9)	51 (56.7)	17 (18.9)	14 (15.5)
A1	4	30	8	8
A2	0	6	1	0
A3	3	15	8	6
B1A1	1	0	0	0

**Table 5:** Contextual characteristics of the injury.

Boat type	Semi rigid	Rigid	Not recall	
Patients (%)	38 53.5	27 38.0	6 8.5	
Position during accident	Sitting	Standing		
Patients (%)	69 97.2	2 2.8		
Localization on the boat	Front	Middle	Rear	
Patients (%)	61 85.9	4 5.6	6 8.5	
Reception	Bottom	Feet	Back	
Patients (%)	63 88.7	2 2.8	6 8.5	
Type of cruise	Tourism	Professional	Transit	
Patients (%)	66 93.0	1 1.4	4 5.6	
Sea activity	Calm	Small swell	Agitated	Not recall
Patients (%)	37 52.1	11 15.5	10 14.1	13 18.3
Type of wave causing the accident	Wave from a boat’s wake	Natural sea wave	Not recall	
Patients (%)	55 77.5	6 8.4	10 14.1	
Wave’s anticipation	Anticipated	Surprise	Not recall	
Patients (%)	15 21.1	52 73.3	4 5.6	
Subjective perception of the boat’s speed	Slow	Moderate	Fast	Not recall
Patients (%)	16 22.5	22 31.0	23 32.4	10 14.1

## Data Availability

Data is available by request to the author.

## References

[1] Ull C., Yilmaz E., Jansen O., Lotzien S., Schildhauer T.A., Aach M., Königshausen M. (2020). Spinal Cord Injury With Tetraplegia in Young Persons After Diving Into Shallow Water: What Has Changed in the Past 10 to 15 Years?. Glob. Spine J..

[2] Ernstbrunner L., Runer A., Siegert P., Ernstbrunner M., Becker J., Freude T., Resch H., Moroder P. (2017). A prospective analysis of injury rates, patterns and causes in Cliff and Splash Diving. Injury.

[3] Donnally C.J., Rothenberg P.M., Metser G., Massel D.H., Butler A.J., Damodar D., Shin S.H., Zakrison T.L. (2018). Orthopedic injuries associated with jet-skis (personal watercrafts): A review of 127 inpatients. Orthop. Traumatol. Surg. Res..

[4] Stanisavljevic S., Irwin R.B., Brown L.R. (1978). Orthopedic injuries in water-skiing: Etiology and prevention. Orthopedics.

[5] Rizzo M.G., Desai S.S., Benson D.C., Vilella F.E., Dodds S.D. (2021). Watercraft propellers as a mechanism of orthopaedic injuries: Injury patterns, management, and complications. Eur. J. Trauma Emerg. Surg..

[6] Neville V., Folland J.P. (2009). The epidemiology and aetiology of injuries in sailing. Sport. Med..

[7] Feletti F., Brymer E., Bonato M., Aliverti A. (2021). Injuries and illnesses related to dinghy-sailing on hydrofoiling boats. BMC Sport. Sci. Med. Rehabil..

[8] Korres D.S., Benetos I.S., Themistocleous G.S., Mavrogenis A.F., Nikolakakos L., Liantis P.T. (2006). Diving injuries of the cervical spine in amateur divers. Spine J..

[9] Carmel A., Drescher M.J., Leitner Y., Gepstein R. (2004). Thoracolumbar Fractures Associated with the Use of Personal Watercraft. J. Trauma Acute Care Surg..

[10] Branche C.M., Conn J.M., Annest J.L. (1997). Personal Watercraft-Related Injuries: A Growing Public Health Concern. JAMA.

[11] Hurpin V., Peyrefitte S., Ruby X., Daniel Y. (2022). Musculoskeletal diseases among French military high-speed boat pilots. Arch. Environ. Occup. Health.

[12] Fradet L., Petit Y., Wagnac E., Aubin C.-E., Arnoux P.-J. (2014). Biomechanics of thoracolumbar junction vertebral fractures from various kinematic conditions. Med. Biol. Eng. Comput..

[13] Yoganandan N., Arun M.W.J., Stemper B.D., Pintar F.A., Maiman D.J. (2013). Biomechanics of human thoracolumbar spinal column trauma from vertical impact loading. Ann. Adv. Automot. Med..

[14] Allami M.K., Drakoulakis E.G., Dinopoulos H., Dunsmuir R., Macdonald D.A., Giannoudis P.V. (2005). Thoracolumbar spinal injuries following speedboat accidents: Is it time to change the safety regulations?. Inj. Extra.

[15] Wild G. (2007). Vertebral wedge fracture after speedboat “splash down”. J. R. Nav. Med. Serv..

[16] Chukwunyerenwa C.K., O’Rourke P. (2010). Compression fractures of the vertebrae during a “bumpy” boat ride. Ir. J. Med. Sci..

[17] Maempel J., Maempel F. (2019). The speedboat vertebral fracture: A hazard of holiday watersports. Scott. Med. J..

[18] Sellier A., Al Falasi M., Joubert C., Desse N., Beucler N., Renard A., Bernard C., Allanic L., Dagain A. (2019). A summer wave of vertebral fractures: The “deck-slap” injury. Acta Neurochir..

[19] Vaccaro A.R., Oner C., Kepler C.K., Dvorak M., Schnake K., Bellabarba C., Reinhold M., Aarabi B., Kandziora F., Chapman J. (2013). AOSpine Thoracolumbar Spine Injury Classification System: Fracture Description, Neurological Status, and Key Modifiers. Spine.

[20] Aslan S., Karcioglu O., Katirci Y., Kandiş H., Ezirmik N., Bilir O. (2005). Speed bump–induced spinal column injury. Am. J. Emerg. Med..

[21] Lane N.E. (2006). Epidemiology, etiology, and diagnosis of osteoporosis. Am. J. Obstet. Gynecol..

[22] Bruno A.G., Burkhart K., Allaire B., Anderson D.E., Bouxsein M.L. (2017). Spinal Loading Patterns From Biomechanical Modeling Explain the High Incidence of Vertebral Fractures in the Thoracolumbar Region. J. Bone Min. Res..

[23] Li W.-J., Guo L.-X. (2020). Influence of different postures under vertical impact load on thoracolumbar burst fracture. Med. Biol. Eng. Comput..

[24] Spurrier E., Gibb I., Masouros S., Clasper J. (2016). Identifying Spinal Injury Patterns in Underbody Blast to Develop Mechanistic Hypotheses. Spine.

[25] Ramasamy A., Hill A.M., Phillip R., Gibb I., Bull A.M.J., Clasper J.C. (2011). The Modern “Deck-Slap” Injury—Calcaneal Blast Fractures From Vehicle Explosions. J. Trauma Acute Care Surg..

[26] Wiker S.F., Miller J.M. (1983). Acceleration Exposures in Forward Seating Areas of Bowrider Recreational Boats. Hum. Factors.

